# Prevalence of the metabolic syndrome in acute myocardial infarction and its impact on hospital outcomes

**DOI:** 10.4103/0973-3930.53120

**Published:** 2009

**Authors:** S. Pandey, N. Baral, S. Majhi, P. Acharya, P. Karki, S. Shrestha, B. K. L. Das, L. Chandra

**Affiliations:** Department of Biochemistry, BP Koirala Institute of Health Sciences, Dharan, Nepal; 1 Department of Medicine, BP Koirala Institute of Health Sciences, Dharan, Nepal; 2 Department of Maulana Azad Medical College, New Delhi, India

**Keywords:** Metabolic syndrome, acute myocardial infarction

## Abstract

**AIMS:**

To ascertain the prevalence of the metabolic syndrome in patients with acute myocardial infarction; to study the impact of the metabolic syndrome on hospital outcomes; and to find out the association of each component of the metabolic syndrome with acute myocardial infarction (AMI).

**SETTING:**

Coronary care unit, Department of Medicine, B P Koirala Institute of Health Sciences, Dharan, Nepal.

**DESIGN:**

Hospital-based cross-sectional study.

**MATERIALS AND METHODS:**

A total of 84 unselected consecutive patients hospitalized with AMI (diagnosed on the basis of WHO criteria) were categorized according to NCEP ATP III criteria.

**STATISTICAL ANALYSIS:**

Data was analyzed by using the Student's t test and Chi-square test.

**RESULTS:**

Among the 84 AMI patients, 22 (26.19%) fulfilled the criteria for metabolic syndrome. Patients with the metabolic syndrome were older (86% were >50 years of age) and females (27%) were more affected than males (25%). In-hospital case fatality was higher in patients having the metabolic syndrome (5/22) than in those without the syndrome (3/62). Among the five components of the metabolic syndrome, the triglyceride levels had the highest positive predictive value (62%) in AMI; this was followed by fasting blood glucose levels (55%).

**CONCLUSION:**

The prevalence of the metabolic syndrome is 26.19%; it is associated with high mortality; among its components, the triglyceride level has the highest positive predictive value in AMI patients.

## Introduction

The metabolic syndrome is one of the major public health issues of this century. It is a constellation of physical conditions and metabolic abnormalities, commonly occurring together, that increases an individual's risk for development of type 2 diabetes mellitus and cardiovascular disease. If the current trend continues, the premature deaths and disabilities resulting from these conditions will increase the financial burden in developed and developing countries.

Several expert groups have attempted to define the diagnostic criteria for the metabolic syndrome. In 1998, the World Health Organization (WHO) proposed a formal definition of the metabolic syndrome; according to this, a person must have either glucose intolerance or insulin resistance along with two of the following four criteria: central obesity, hypertension, dyslipidemia, and albuminuria.[1] In 2001, the National Cholesterol Education Program Adult Treatment Panel III (NCEP ATP III) provided a new definition for the metabolic syndrome, according to which a person must have three of the following five abnormalities: abdominal adiposity, hypertension, hypertriglyceridemia, low high-density lipoprotein cholesterol, and elevated fasting glucose.[2] Based on the definitions of NCEP ATP III, a cross-sectional study conducted by the Third National Health and Nutrition Examination Survey in a US population found that the prevalence of the metabolic syndrome was 25% among white Americans; the prevalence was 44% among those 50 years and older.[3] More recent estimates of the prevalence of the metabolic syndrome ranged from 21.3–32.8% among the participants in the Framingham Offspring Study and the San Antonio Heart Study.[4] Moreover, a recent study in patients with established coronary artery disease or stroke showed that the prevalence of the metabolic syndrome correlated with the extent of vascular damage.[5] Little is known about the prevalence of the metabolic syndrome in patients with acute coronary syndrome, particularly in South-East Asians. There is also limited information available on the impact of the metabolic syndrome on hospital outcomes after presentation for an acute myocardial infarction (AMI) in South-East Asians.

The present study aims to ascertain the prevalence of the metabolic syndrome in patients with acute myocardial infarction, to study the impact of the metabolic syndrome on hospital outcomes, and to find out the association of each component of the metabolic syndrome with AMI.

## Materials and Methods

This hospital-based, cross-sectional study was conducted in the Department of Biochemistry, in collaboration with Department of Medicine, B.P. Koirala Institute of Health Sciences, Dharan, Nepal. This institute, named after the great nationalist and former Prime Minister of Nepal, is a 646-bedded tertiary care teaching hospital and university in the eastern part of Nepal. It has a coronary care unit under the Department of Medicine. The study was carried out from March 2005 to July 2006. One hundred and twenty patients suspected of having acute myocardial infarction (AMI) were screened. Eighty-four patients, attending emergency and admitted in the coronary care unit, who were diagnosed as having AMI as per the WHO criteria, were included in the study with their consent. Blood samples were drawn at admission for creatine kinase-total and creatine kinase-2. The following morning, samples for triglyceride and HDL-C were collected, and on day 3, a sample for estimation of blood glucose level was collected. All the biochemical parameters were assayed on Vitalab Selectra-E–Clinical Chemistry Analyzer. Waist-circumference was measured and blood pressure was recorded in all subjects.

Patients were diagnosed as having the metabolic syndrome if they had any three of the following five components: abdominal obesity (waist circumference >102 cm in men and >88 cm in women), high triglyceride levels (≥150 mg/dl), low HDL-C levels (<40 mg/dl in men and <50 mg/dl in women), elevated fasting glucose levels (≥110 mg/dl), and high blood pressure (treated hypertension and systolic blood pressure/diastolic blood pressure ≥130/85 mm Hg). Data was analyzed using the Student's t test and the Chi-square test.

## Results

**Figure 1:**
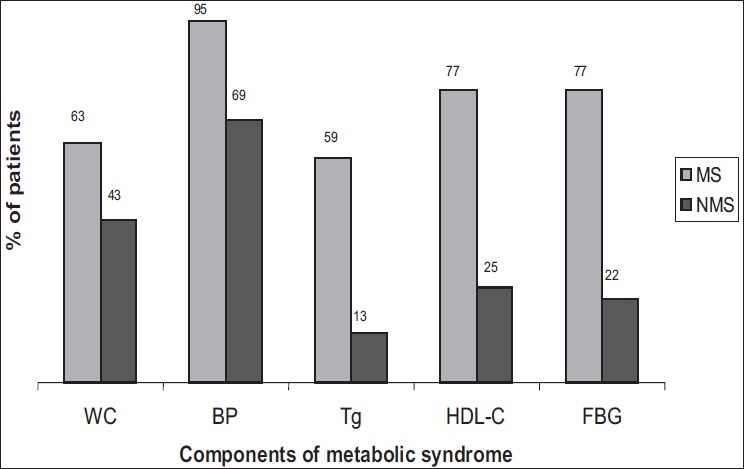
Prevalence of each component of the metabolic syndrome in AMI cases having metabolic syndrome (MS) and nonmetabolic syndrome (NMS). WC: increased waist circumference, BP: high blood pressure, Tg: elevated triglyceride levels, HDL-C: low high density lipoprotein cholesterol levels, FBG: elevated fasting blood glucose

Eighty-four patients with a confirmed diagnosis of AMI as per WHO criteria were enrolled with their consent. Among these patients, 22 (26.19%) had the metabolic syndrome according to the NCEP ATP III criteria. The prevalence of the components of the metabolic syndrome and nonmetabolic syndrome is shown in Figure 1.

The clinical characteristics of the study population are shown in Table 1. The majority of the patients with the metabolic syndrome had more than three of the components. Females were affected more than males. Among AMI patients, the majority of those with the metabolic syndrome (*n* = 19/22) were more than 50 years of age. Among the younger patients (40–49 years) with AMI, there was a significantly higher proportion (75%) with the metabolic syndrome.

**Table 1:** The clinical characteristics of the study population

Characteristics		Patients without Metabolic Syndrome (n=62)	Patients with Metabolic Syndrome (n=22)	*P* value
Demographics and risk factors				
Age (year)		60.69 ± 11.68	61.40 ± 10.09	NS
Sex	Male	41	14	
	Female	21	08	NS
Smoker		46	12	
Non smoker		16	10	NS
Vegetarian Diet		13	06	
Nonvegetarian Diet		49	16	NS
Alcoholic		19	07	
Nonalcoholic		43	15	NS
Components				
Increased Waist Circumference		27	14	NS
High Blood Pressure		43	21	NS
Elevated Triglycerides		08	13	0.001
Low HDL-Cholesterol		16	17	0.001
Elevated Fasting Glucose		14	17	0.001
In-Hospital Outcome				
Hospital stay (median days)		04	05	NS
Mortality		03	05	NS

The in-hospital case fatality rate among AMI patients with the metabolic syndrome was more (5/22) than that of patients without the syndrome (3/62). The median hospital stay among patients with the metabolic syndrome was four days *vs* five days for those without the syndrome; however, this difference was not statistically significant.

Among the five components of the metabolic syndrome, the triglyceride level had the highest positive predictive value (62%) for AMI, and was followed by fasting blood glucose levels (55%), decreased HDL-C levels (51.5%), waist circumference (34.14%), and blood pressure (33%).

## Discussion

To the best of our knowledge, this is the first study on the association and impact of the metabolic syndrome and its components on AMI cases at the tertiary care hospital and medical institute in Nepal. Results from the National Health and Nutrition Survey III, which was conducted from 1988 to 1994, showed that the unadjusted prevalence rate of the metabolic syndrome among adults aged 20 years or older was 21.8% and the age-adjusted prevalence was 23.7%.[3] The prevalence peaked at 43.5% among persons aged 60–69 years and was 42% among participants aged 70 years or older. A randomly selected population of six clusters in the city of Jaipur, India, found the prevalence of the metabolic syndrome as being 7.9% in males and 17.5% in females.[6]

There is very limited information about the relationship of the metabolic syndrome with AMI, particularly in South-East Asia, although Western studies suggest that it is very commonly associated with coronary artery disease.[7]

In this study, 26.19% cases of AMI cases had the metabolic syndrome; this was mainly in females and the older age-groups. A similar high (46%) prevalence of the syndrome in AMI patients among females and older age-groups has also been reported by Zeller *et al*.[7]

In the present study, in-hospital fatality was more among those with the metabolic syndrome (5/22) than those without the syndrome (3/62). Another study has also reported that the metabolic syndrome was associated with an increased case fatality rate.[8] However, after adjusting for the major determinants of mortality in AMI, the metabolic syndrome was not seen to be an independent predictor of this.[7]

Among the individual components of the metabolic syndrome, we found that raised triglyceride levels had the highest positive predictive value (62%) and this was followed by fasting blood glucose (55%); blood pressure had the least positive predictive value. This indicates that high triglyceride levels are associated with higher morbidity and may predispose to a higher mortality. As reported by a previous study,[9] at long-term follow up, hypertension is only a modest predictor of death.

The present study reveals the high prevalence of the metabolic syndrome in AMI cases, particularly in females and older patients. Patients having the metabolic syndrome have higher in-hospital fatality. Of the individual components of the metabolic syndrome, triglyceride levels had the highest positive predictive value in AMI cases and it was followed by fasting hyperglycemia.

As the prevalence of the metabolic syndrome is high worldwide and increasing day by day due to sedentary lifestyles, the findings of the present study has important implications for clinical practice. Emphasis must be placed on the intake of balanced diets and the control of blood lipid levels, particularly that of triglycerides. Since elevated fasting blood glucose has such a high predictive value in AMI, there must be a careful search for deranged carbohydrate metabolism in all cases.
